# A154 COMPARING RESPONSE TO INTRAVENOUS IRON INFUSION IN CROHN’S DISEASE AND ULCERATIVE COLITIS

**DOI:** 10.1093/jcag/gwab049.153

**Published:** 2022-02-21

**Authors:** S Su, J Stone, C N Bernstein

**Affiliations:** 1 Gastroenterology, University of Manitoba, Winnipeg, MB, Canada; 2 Uiversity of Manitoba, Winnipeg, MB, Canada

## Abstract

**Background:**

Iron deficiency anemia (IDA) is common in persons with inflammatory bowel disease (IBD). Current evidence-based guidelines suggest iron replacement therapy in IBD patients with IDA. Intravenous (IV) iron has been demonstrated to be more effective than oral iron replacement in the IBD population, and this is thought to be related to oral iron being poorly tolerated, absorbed, and possibly having an adverse impact on the gut microbiome. Studies have not directly compared the response of IV iron between persons with ulcerative colitis (UC) and Crohn’s disease (CD).

**Aims:**

(1) To compare the increase in serum hemoglobin and ferritin following IV iron therapy between persons with UC and CD. (2) To determine factors associated with response to IV iron (other than disease type), including age, sex, IBD therapies, abdominal surgeries, and IBD phenotype.

**Methods:**

In a retrospective chart review, we evaluated 536 IV iron infusions (iron sucrose) prescribed to 117 IBD patients by a single gastroenterologist between 2012–2020, and collected data on IBD type, age, sex, medications (IBD therapies, NSAIDs, ASA, oral iron), abdominal surgeries, and IBD phenotype. Statistical analysis was performed using SPSS version 26.

**Results:**

Most IV iron infusions were given to patients with CD (77% of infusions, 68% of persons). The majority of infusions were given as a series of multiple iron infusions (84%) over a mean of 27 weeks, rather than a single infusion. Persons with UC had a greater increase in serum ferritin than those with CD (mean difference ± SE of 13.2 ± 5.6 µg/L, p = 0.02). There was no significant difference in the increase in serum hemoglobin between UC and CD (UC= 6.5 ± 1.0 g/L; CD 4.9 ± 2.1 g/L; p = 0.62).

**Conclusions:**

Persons with UC had a better ferritin response to IV iron therapy than persons with CD. Patients with UC were prescribed less IV iron than those with CD. In summary, persons with CD may require greater dosing of IV iron therapy than patients with UC. Further studies are needed to discern if this difference is secondary to CD being associated with a greater extent of mucosal disease burden, impaired iron absorption, or a greater intolerance to oral iron.

**Funding Agencies:**

Fellowship Funding from Pfizer Canada

